# Canaliculops Mimicking Canaliculitis

**DOI:** 10.7759/cureus.74060

**Published:** 2024-11-20

**Authors:** Chrisha Faye T Habaluyas, Jonnah Kristina C Teope, Mayari Ito, Yasuhiro Takahashi

**Affiliations:** 1 Oculoplastic, Orbital & Lacrimal Surgery, Aichi Medical University Hospital, Aichi, JPN

**Keywords:** canaliculitis, canaliculops, common canalicular obstruction, dacryoendoscopy, intracanalicular granulation tissue

## Abstract

Canaliculops is a rare case of a medial canthal mass due to a non-infectious dilation of the canaliculus and easily mimics canaliculitis with canalicular dilation. We report a case of a 68-year-old woman with a five-year history of progressive swelling of the right upper eyelid. The patient was previously diagnosed with canaliculitis at other clinics and had a history of diabetes mellitus. A purplish-red cystic lesion in the right upper eyelid, medial to the punctum, was noted. However, the lesion was non-tender. Dacryoendoscopic examination showed an expanded right upper horizontal canaliculus with minor inflammatory changes and without intracanalicular granulation tissue. Right common canaliculus obstruction was managed with dacryoendoscopic probing and lacrimal tube insertion. Cytodiagnostic examination of accumulated fluid in the dilated lacrimal canaliculus showed a few foam and inflammatory cells. At three-month follow-up, the lacrimal tube was removed, and no recurrence of epiphora was noted.

## Introduction

Canaliculops is a rare entity of a non-inflammatory dilatation of the canalicular wall caused by obstruction of both the punctum and common canaliculus [1-3]. Most cases are acquired, but congenital canaliculops, often associated with punctal agenesis, has also been described [4]. The majority of reported cases share common characteristics, including clinical chronicity, absence of tenderness on palpation, and lack of inflammation [3]. These cases are typically noted in middle-aged individuals with mostly female preponderance [3]. Ectatic changes of the horizontal canaliculus in canaliculops can easily be misdiagnosed as canaliculitis with canalicular dilation [5]. We herein report a case of canaliculops initially misdiagnosed as canaliculitis.

## Case presentation

A 68-year-old Japanese woman presented with a 5-year history of progressive swelling at the medial end of the right upper eyelid. This symptom gradually deteriorated, and the patient had epiphora a few months before referral to our hospital. Ophthalmologists at other clinics suspected canaliculitis and consulted with us. The patient did not receive topical treatment but was instead provided with nutritional therapy for diabetes mellitus (glycated hemoglobin, 6.2%). Her family history was unremarkable.

**Figure 1:**
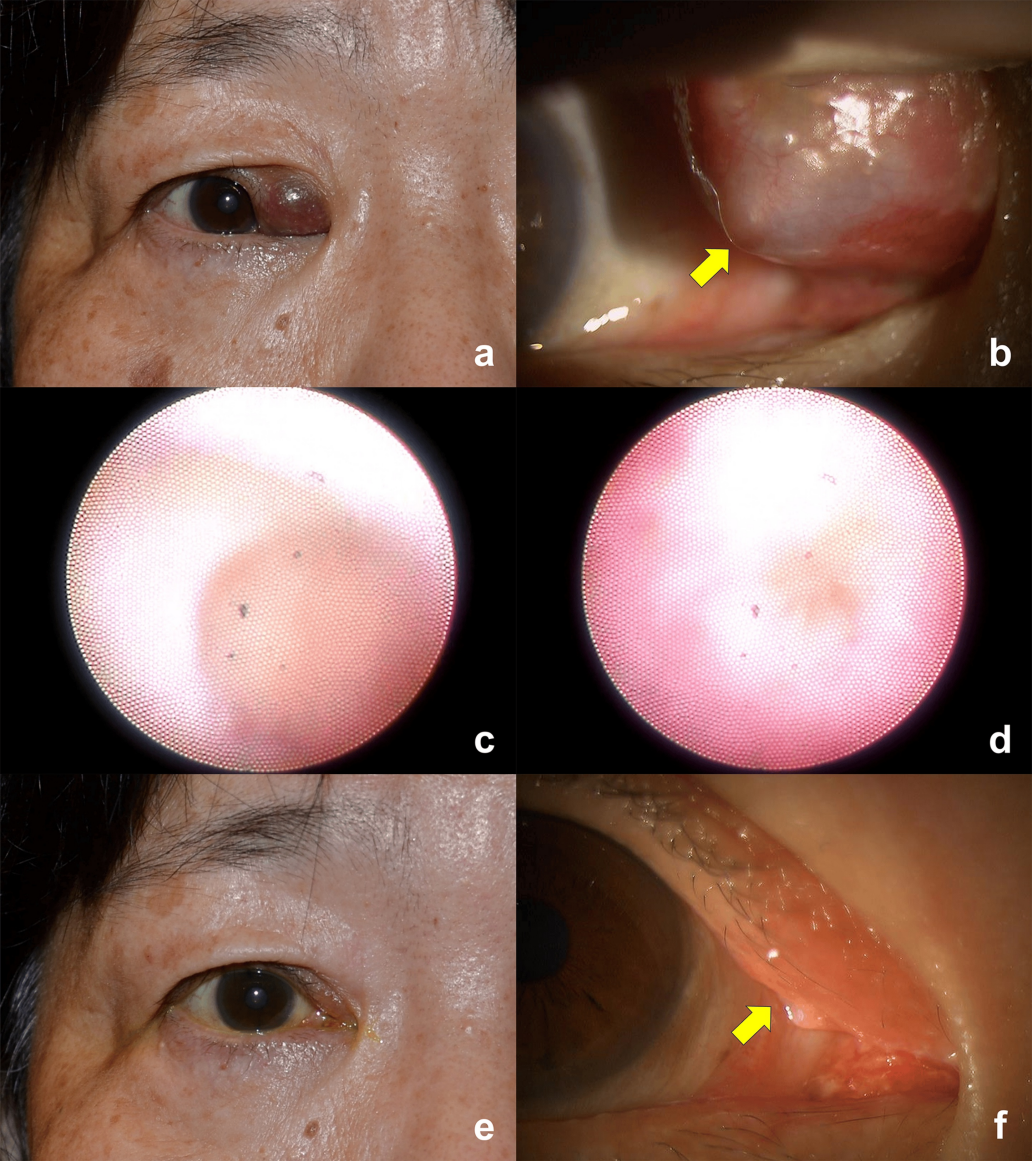
Facial photographs and dacryoendoscopic images (A) A face photo was taken during the initial examination, showing a purplish-red cystic lesion in the right medial canthal region. (B) A slit-lamp examination showed obstruction of the right upper punctum. Dacryoendoscopic examination showed dilation of the horizontal canaliculus without granulation tissue (C) and common canalicular obstruction (D). (E) A face photo taken three months after surgery showed deflation of the lesion. (F) A slit-lamp examination showed a patent right upper punctum (arrow).

On initial evaluation, a slit-lamp examination of the right eye revealed a purplish-red cystic lesion in the eyelid, medial to the right upper punctum (Figures 1A, 1B). However, the lesion was non-tender. The right upper punctum was obstructed (Figure 1B). Blood tests revealed no elevation of white blood cell count and C-reactive protein.

Surgery was performed under local anesthesia. After punctoplasty, translucent cyst fluid was drained, aspirated, and collected for cytodiagnosis. Dacryoendoscopic examination showed an expanded right upper horizontal canaliculus with minor inflammatory changes and without intracanalicular granulation tissue (Figure 1C). Since the right common canaliculus was obstructed (Figure 1D), direct dacryoendoscopic probing was performed to open the common canaliculus. A bicanalicular lacrimal tube was inserted.

Cytodiagnostic examination showed a few foam and inflammatory cells. At three-month follow-up, the lacrimal tube was removed. The cystic lesion deflated (Figure 1E), and the right upper punctum was patent (Figure 1F). The patient had no symptoms of epiphora.

## Discussion

We report a rare case of canaliculops mimicking canaliculitis. Being a rare disorder, ophthalmologists may be unfamiliar with the condition, leading to a high likelihood of initial misdiagnosis [6]. 

Canaliculops is characterized by painless dilation of the canaliculus due to serous fluid accumulation [3,7]. Key features include fluctuant, bluish or translucent medial eyelid swelling without tenderness or discharge [3,8]. Lokdarshi et al. emphasized simple tests such as fluctuation and transillumination to identify these cystic lesions [8], while diagnostic imaging tools such as ultrasound biomicroscopy and optical coherence tomography provided detailed insights [4,7].

A purplish-red cystic lesion was observed medial to the right upper punctum and was initially diagnosed as canaliculitis with canalicular dilation. Our patient was being treated for diabetes mellitus, which is a predisposing factor for canaliculitis [9]. However, canaliculitis typically involves tenderness, which is absent in this case. Cytodiagnostic results of the non-mucopurulent cyst showed a few inflammatory cells without bacteria. The few inflammatory cells, in this case, may be attributed to the chronicity of the lesion. Moreover, dacryoendoscopic examination revealed no granulation tissue in the dilated horizontal canaliculus [5,9-11]. A common canalicular obstruction is a risk factor for the development of canaliculitis but is not essential for the development [11]. Although histopathological confirmation of positive CK7 and CK17 immunostaining was not noted in the canalicular lesion [3], the clinical findings in our patient corresponded to canaliculops.

Treatment depends on the underlying cause [6,12]. Acquired cases are typically resolved through minimally invasive methods. In contrast, congenital cases often require more complex interventions. Bothra et al. demonstrated that creating a new punctum and intubation could restore anatomical patency, though mild functional limitations may persist [4]. Marsupialization remains a viable option for localized cases [2,3,8].

In our case, punctoplasty was performed, and bicanalicular lacrimal tube was inserted using direct dacryoendoscopy to manage the patient. Previous case reports showed that dacryoendoscopy enables probing of the obstructed area, which is more reliable and less invasive than other procedures [1,2]. Currently, the patient’s symptoms of epiphora and eyelid swelling resolved after the procedure, affirming dacryoendoscopy as a reliable method.

## Conclusions

To conclude, in cases of canaliculops, a thorough history and meticulous clinical examination should be made to identify this rare entity. The absence of specific immunostaining results does not rule out a clinically evident case of canaliculops. Canaliculops should be included in the differential diagnosis of cystic lesions at the medial canthus to avoid misdiagnosis and provide the appropriate treatment.
